# Comparative transcriptome analysis reveals osmotic-regulated genes in the gill of Chinese mitten crab (*Eriocheir sinensis*)

**DOI:** 10.1371/journal.pone.0210469

**Published:** 2019-01-10

**Authors:** Zhigang Yang, Junyu Zhou, Banghong Wei, Yongxu Cheng, Long Zhang, Xiaomin Zhen

**Affiliations:** 1 Key Laboratory of Freshwater Aquatic Genetic Resources, Ministry of Agriculture, Shanghai Ocean University, Shanghai, China; 2 Centre for Research on Environmental Ecology and Fish Nutrition (CREEFN) of the Ministry of Agriculture, Shanghai Ocean University, Shanghai, China; 3 National Demonstration Center for Experimental Fisheries Science Education, Shanghai Ocean University, Shanghai, China; Kaohsiung Medical University, TAIWAN

## Abstract

Salinity is one of the most important abiotic factors directly affecting the reproduction, molting, growth, immune, physiological and metabolic activities of Chinese mitten crab (*Eriocheir sinensis*). This species has strong osmoregulatory capacity and can maintain stringent internal homeostasis. However, the mechanisms conferring tolerance to salinity fluctuations are not well understood. To reveal the genes and pathways involved in osmoregulation, adult male crabs (body weight = 110 ± 5 g) were acclimated for 144 h in freshwater (FW, 0 ppt) or seawater (SW, 25 ppt). Changes in the transcriptome of crab gills were then analysed by RNA-Seq, and 174,903 unigenes were obtained. Comparison of genes between FW- SW-acclimated groups identified 932 genes that were significantly differentially expressed in the gill, comprising 433 and 499 up- and downregulated transcripts. Gene Ontology functional enrichment analysis revealed that important biological processes related to salt stress were significantly enriched, including energy metabolism, ion transport, signal transduction and antioxidant activity. Kyoto Encyclopaedia of Genes and Genomes enrichment analysis mapped the differentially expressed genes to 241 specific metabolic pathways, and pathways related to energy metabolism, oxidative phosphorylation and the tricarboxylic acid (TCA)/citrate cycle were significantly enriched. Salinity stress altered the expression of many enzymes involved in energy metabolism, ion transport, signal transduction and antioxidant pathways, including citrate synthase (CS), Na^+^/K^+^-ATPase (NKA), Na^+^-K^+^-2Cl cotransporter-1 (NKCC1), dopamine receptor D1 (DRD1), synaptic binding protein 1 (STXBP1), Cu^2+^/Zn^2+^ superoxide dismutase (SOD1) and glutathione S-transferase (GST). Additionally, the obtained transcriptomic sequencing data provided a useful resource for identification of novel genes, and further physiological analysis of Chinese mitten crab.

## Introduction

Chinese mitten crab (*Eriocheir sinensis*) is one of the most economically important species for freshwater aquaculture in China, from the Eastern Pacific coast to the Korean Peninsula [1]. This freshwater crab species is native to China, but can also be found as an invasive species in Europe and America [2]. After puberty molting, sexually mature crabs migrate downstream to brackish water for reproduction, and juvenile crabs migrate from the sea to freshwater to grow into adult crabs [3, 4]. *E*. *sinensis* is a euryhaline species and a strong osmoregulator [5, 6]. During reproductive and growth migrations, *E*. *sinensis* can adjust its hemolymph osmotic concentration to adapt the changes in environmental salinity [7].

A number of studies have shown that salinity affects energy metabolism, ion transport, signal transduction and oxidative stress in juvenile and adult crabs [8, 9, 10, 11, 12]. A lot of energy is consumed to regulate osmotic pressure and ionic balance in response to changes in environmental salinity. At the isosmotic point, the pressure of infiltration is minimal and crustaceans can regulate homeostatic osmotic pressure using minimal energy consumption, whereas more energy is needed for osmotic adjustment [13, 14]. However, the underlying mechanisms of energy metabolism related to osmoregulation in *E*. *sinensis* are poorly understood, especially at the molecular level, and the genes and pathways involved remain largely unknown.

Inorganic ions and several specific free amino acids such as glycine, proline and alanine are the main contributors to hemolymph osmolality in crustaceans [6, 11, 15]. Ion transport is mainly achieved by the action of various ion transport enzymes such as Na^+^/K^+^-ATPase (NKA), V-type H^+^-ATPase (VHA), carbonic anhydrase and HCO_3_^—^ATPase [16, 17, 18]. Ion exchange mainly occurs in the posterior gill [19, 20]. However, studies on ion transporters in *E*. *sinensis* have mainly focused on changes in ion transport enzyme activity, and few studies have been carried out at the molecular level in crustaceans.

The osmotic adjustment process in aquatic animals is complicated [21]. Previous studies have demonstrated that in order to cope with salinity stress, aquatic animals can activate signal transduction pathways to maintain osmotic homeostasis [22, 23, 24]. Studies have also shown that NKA can be activated by the cAMP-dependent pathway to adjust osmotic pressure under salinity stress [9]. However, information about the signal transduction events controlling osmoregulation in aquatic animals is limited, and further work is needed to remedy this [23]. Many studies have shown that salinity stress can increase reactive oxygen species (ROS) in aquatic animal tissues, resulting in oxidative damage [25, 26]. In response, antioxidant systems are stimulated to scavenge ROS [10]. However, the underlying mechanisms of ROS generation induced by salinity stress are unclear.

Transcriptomics approaches have been widely applied to study osmotic adjustment in aquatic animals [12, 27, 28, 29]. Advances in transcriptome technology have greatly improved our ability to analyse non-model species. Therefore, in order to further investigate the potential mechanisms of osmotic regulation in *E*. *sinensis* at the transcriptional level, we performed transcriptome sequencing analysis of osmoregulation and physiological responses in *E*. *sinensis* exposed to seawater conditions, with an emphasis on energy metabolism, transporters, signal transduction and antioxidants.

## Materials and methods

### Animals and experimental conditions

Chinese mitten crabs (body weight = 110 ± 5 g) were obtained from the Chongming Research Base of Shanghai Ocean University and kept in a freshwater tank for 1 week for acclimation. After acclimation, healthy crabs deprived of food for 24 h were randomly divided into a freshwater group FW (0 ppt) and a seawater group SW (25 ppt) in six tanks (80 cm × 40 cm × 40 cm), with 10 crabs in each tank, and all treatments were performed in triplicate. The details of the experiment were deposited in protocols.io. http://dx.doi.org/10.17504/protocols.io.v4ve8w6 [PROTOCOL DOI]

There was no feeding during the experiment, which had a 12 h light / 12 h dark photoperiod. Water quality parameters were monitored twice daily to maintain conditions of 24.5–30.0°C, pH 8.0 ± 0.4, dissolved oxygen >5 mg/L, and total ammonia nitrogen <0.01 mg/L. After salinity stress for 144 h, six crabs per tank were treated with tricaine methane sulfonate (MS 222, 200 mg/L) and the three posterior gill tissues were harvested immediately and stored at -80°C for RNA extraction.

### Hemolymph osmolarity measurement

After acclimation, healthy crabs were selected and transferred to tanks (80cm×40cm×40cm) for experiment. A salinity group SW (ppt 25) and a control group FW (ppt 0) were set up, 10 crabs were placed in each tank and all treatments were performed in triplicate. Samples were taken at 0, 3, 6, 12, 24, 48, 72, 96 and 144 hours of the experiment. At each sampling, three crabs were taken from each experimental group. Crabs were placed on ice for 15 minutes for anesthesia. Hemolymph samples were extracted from the base joints of the third foot with a 2 ml syringe. Hemolymph osmolarity was measured by OSMOMAT 3000 (Gonotec). The experimental data were analyzed by SPASS 18.0 software, and *p*-value <0.05 was significant difference.

### RNA extraction, transcriptome library preparation, and RNA-Seq

Total RNA was extracted from gill tissue using TRIzol (Invitrogen) according to the manufacturer’s instructions. The concentration and quality of total RNA were examined using Agilent 2100 and NanoDrop 2000 instruments prior to subsequent experiments. Only high-quality RNA samples with optical density (OD) 260/280 values >1.8 and <2.2, a 28S:18S ratio >1.0, and RNA yield >5 μg were used for subsequent transcriptome analysis.

An Illumina TruseqTM RNA sample prep kit was used to construct an RNA-Seq transcriptome library. PolyA mRNA was separated from total RNA using magnetic beads with Oligo (dT). Upon addition of fragmentation buffer, enriched mRNA can be randomly broken into small fragments of ~200 bp. First-stand complementary DNA (cDNA) was synthesised using random hexamer primers with mRNA as template. Subsequently, second-strand cDNA was synthesised and a stable double-stranded structure was formed. End Repair Mix (Illumina) was used to repair cohesive ends of second-strand cDNA, and a single A base was added at the 3’ end for adapter ligation. Fifteen cycles of PCR amplification were carried out to enrich the cDNA library. Target bands were recovered from 2% agarose gel electrophoresis experiments (Certified Low Range Ultra Agarose). TBS-380 was used to quantify cDNA, and cDNA fragments were amplified to clusters by bridge PCR. Finally, a HiSeq4000 platform (Illumina) was used for sequencing.

### *De novo* assembly of sequencing reads

Raw data arising from Illumina sequencing were firstly quality-trimmed and adapter-clipped using SeqPrep (https://github.com/jstjohn/SeqPrep) and Sickle (https://github.com/najoshi/sickle). In this process, reads containing adapter and poly-N sequences were removed, and reads with a quality percentage (Q value) <5 bases higher than 50% were also removed. The resulting clean data were used for RNA *de novo* assembly with Trinity (http://trinityrnaseq.sourceforge.net/, Version: trinityrnaseq-r20140413). All sequence reading segments were assembled from scratch to generate overlapping groups and single sequences. This analysis was the basis for follow-up and biological functional analyses.

### Functional annotation of transcripts

Transcripts obtained from gills subjected to different salinity treatments were scanned against NCBI non-redundant (Nr) protein, NCBI nucleotide (Nt), Swiss-Prot, and Kyoto Encyclopaedia of Genes and Genomes (KEGG) databases using BLASTx (version 2.2.25) with an E-value cut-off of <1.0×10^−5^. Blast2GO (http://www.Blast2go.com/b2ghome, version2.5.0) was used to obtain Gene Ontology (GO) annotation information, and the annotation results were classified into biological processes, molecular functions, and cellular components. Pathways in which transcripts were involved were analysed by KEGG (http://www.genome.jp/kegg/), a database that integrates genomic, chemical, and functional information.

### Differential gene expression and enrichment analysis

Differentially expressed genes (DEGs) were identified based on read count data obtained from the analysis of gene expression levels. Gene expression levels were measured according to the Fragments Per Kilobase of transcript per Million fragments mapped (FPKM) method. Trimmed means of M-values (TMM) was used to standardise read count data, and differences were analysed using the DEGseq package for R. Filtering thresholds were q-value <0.005 and |log2FoldChange| >1. Cluster analysis was performed based on DEG levels. GO enrichment analysis of DEGs was performed using GOseq based on the Wallenius non-central hypergeometric distribution. This approach allows the probability of GO terms enriched for different genes to be more accurately calculated.

### Quantitative real-time PCR (qRT-PCR)

Ten DEGs were randomly selected for validation of the Illumina sequencing data by qRT-PCR using primers listed in Table 1. RNA from gill tissue was extracted from FW and SW groups according to the manufacturer’s instructions. After reverse-transcription, primers were used to carry out qRT-PCR. The 18S rRNA gene was used as an internal reference. First-stand cDNA was synthesised from 1 μg of RNA using a PrimeScrip RT reagent kit with gDNA Eraser (Cat. No. RR036A; TaKaRa). The qRT-PCR reaction system contained 10 μL 2×SYBR Premix Ex Taq (Cat. No. RR420A; TaKaRa), 0.2 μmol/L primers. and 2 μL cDNA template. Reactions were performed on an ABI 7500 Real-Time PCR System (Life Tech, Applied Biosystems). There were six biological repeats at each data point and each sample was repeated in triplicate in order to reduce PCR system error. The 2 –^ΔΔCt^ method was employed to determinate relative expression levels for each sample.

**Table 1:** Primers used for qRT-PCR.

Genes	Primers (5’–3’)
*NDUFV1*	F: TTGGACAAGGGTGTCGACTG R: CAGTGGGAAATCCAGCACCT
*GAPDH*	F: GACATTGTGCTCTCCAACGC R: CGACGAGGGGATGATGTTGT
*ACSBG*	F: CAGGGAGGGAGATTCTTGCG R: ATACGATTTGGTCCTCGGGC
*ABCC3*	F: AAACCCGACGACTCAACTGT R: GCCAATGTCTGCAAAATCTACT
*DRD1*	F: GAGAGACGCTTCCAACCTCC R: CCAGCGACTCCAGAGTTACC
*ARF1*	F: CTGCATCAGTATCGACCGCT R: GTAGGCCATGTTCACCACGA
*SOD1*	F: TTGGAGATGGTCCCCTCGAT R: AACTACCCCTCGCTGCTCTA
*MIF1*	F: GAATGCGACCGCAGAATGAG R: CCCACCATATGAGGCATCGG
*NKCC1*	F: GGTTAAGCACGTGGTGAGGA R: TCCTTGGCTTCAATGCCGAT
*ATP1B*	F: CACGTGACTGAGTGTTCCGA R: ATGCATGGGGATTCACGGTT
*NKA*	F: TGAATGACTCCCCAGCTCTCAAG R: CAGAATCATGTCAGCAGCCTGCT
*18S*	F: GGGTCCGAAGCGTTTACT R: TCACCTCTAGCGGCACAA

NDUFV1, NADH dehydrogenase (ubiquinone) flavoprotein 1; GAPDH, Glyceraldehyde 3-phosphate dehydrogenase; ACSBG, Long-chain-fatty-acid-CoA ligase; ABCC3, Canalicular multispecific organic anion transporter 2; DRD1, Dopamine receptor D1; ARF1, ADP-ribosylation factor 1; SOD1, Cu2^+^/Zn2^+^ Superoxide dismutase-1; MIF1, Macrophage migration inhibitory factor; NKCC1, Na^+^-K^+^-2Cl cotransporter-1; ATP1B, Sodium/potassium-transporting ATPase subunit beta; NKA, Na^+^/K^+^ATPase.

## Results

### Hemolymph osmolarity of *E*.*sinensis*

**Fig 1:**
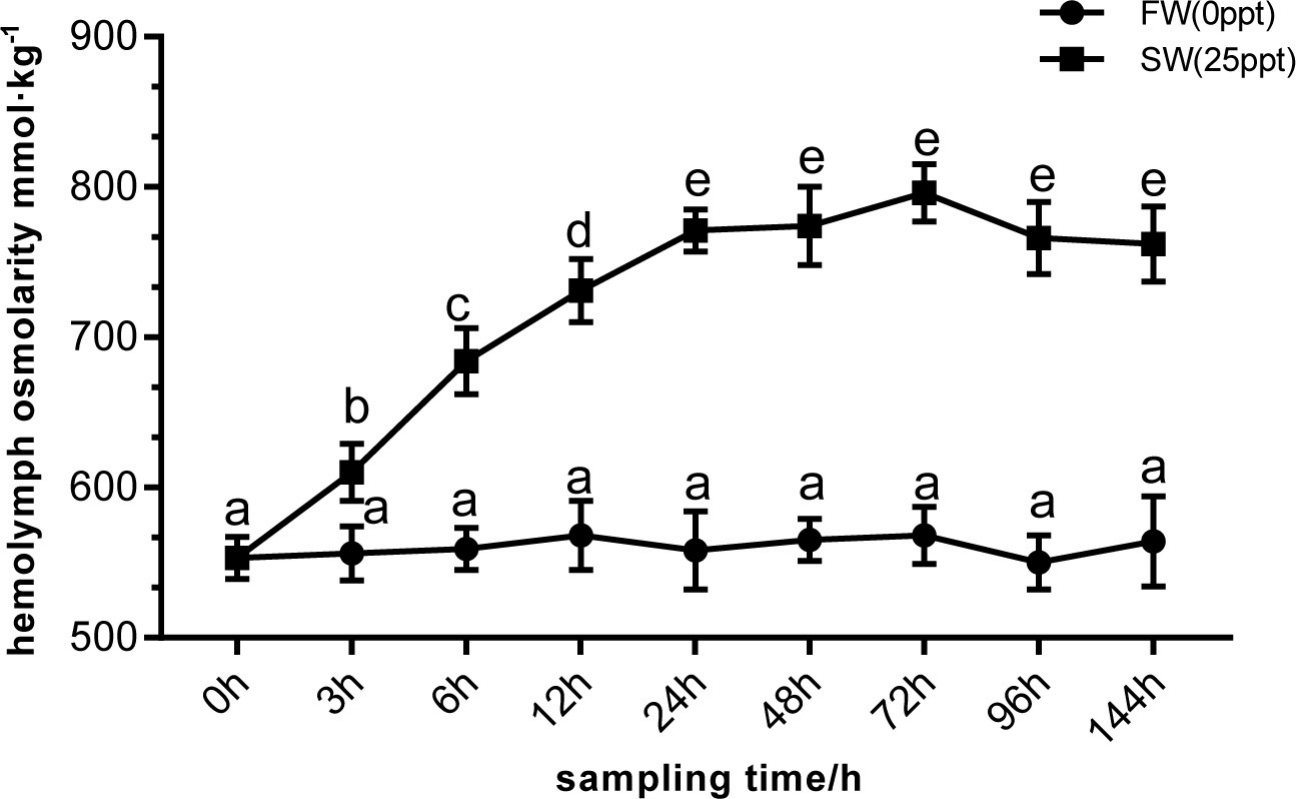
Hemolymph osmolarity of *E*.*sinensis* at different time points under high salinity stress. The different letters mean significant differences.

The hemolymph osmolarity of crabs in SW group was higher than that of control group FW at corresponding sampling time. Hemolymph osmolarity in SW group increased rapidly from 0 hour to 24 hour, with the effect of salinity, hemolymph osmolarity first increased and then decreased. Hemolymph osmolarity decreases after reaching the highest point at 72 hour (Fig 1).

### Sequencing and *de novo* assembly

A total of 47,446,444 raw reads were generated from *E*. *sinensis* gill tissue. After removing low-quality sequences and adapter clipping, 45,517,342 clean reads remained, including 24.1 million clean reads from FW samples and 21.4 million clean reads from SW samples (Table 2). Using Trinity for *de novo* assembly, 210,835 transcripts were obtained. The longest transcript of each gene was taken as a unigene for subsequent analysis, and 174,903 unigenes were obtained. This process yielded 164,908 transcripts (78.22%) and 144,562 unigenes (82.65%) ranging from 200 bp to 500 bp in length, and the average length of transcripts and unigenes was 494 bp and 433 bp, respectively (Table 3; S1 Fig).

**Table 2:** Statistics for Illumina short reads from the *E*. *sinensis* gill transcriptome.

Sample name	Raw reads	Clean reads	Q20 (%)^a^	Q30 (%)^b^	GC Content (%)^c^
FW1	25,161,634	24,136,230	96.39	92.28	50.77
SW1	22,284,810	21,381,112	96.81	92.86	53.10
Total	47,446,444	45,517,342			

^a^ Q20%, percentage of bases with Phred value >20.

^b^ Q30%, percent of bases with Phred value >30.

^c^ GC%, percentage of G and C bases among total bases.

**Table 3:** Summary of RNA-Seq *de novo* assembly results.

	Min length	Mean length	Max length	N50^a^	N90^b^	Total nucleotides
Transcripts	201	494	15,986	615	230	104,198,495
Unigenes	201	433	15,986	456	224	75,751,538

^a^N50, Transcripts sorted by length from long to short, for which the sum of the length of transcripts was not less than 50% of the total length of mosaic transcripts.

^b^N90, Transcripts sorted by length from long to short, for which the sum of the length of transcripts was not less than 90% of the total length of mosaic transcripts.

### Annotation and functional analysis of gill transcripts

Seven databases, namely Nr, Nt, Protein family (PFAM), Clusters of Orthologous Groups (COG), SwissProt, KEGG, and GO, were used to obtain comprehensive gene functional information. A total of 28,296 (16.17%) unigenes were matched using the NR database, 12,448 (7.11%) unigenes were matched using the NT database, 21,985 (12.56%) unigenes were matched using the SwissProt database, and 44,672 (25.54%) unigenes were annotated in at least one database (Table 4).

**Table 4:** Summary of *E*. *sinensis* gill transcriptome annotation information.

Category	Number of unigenes	Percentage (%)
Annotated in NR	28,296	16.17
Annotated in NT	12,448	7.11
Annotated in KO	13,347	7.63
Annotated in SwissProt	21,985	12.56
Annotated in PFAM	30,611	17.5
Annotated in GO	30,578	17.48
Annotated in COG	15,241	8.71
Annotated in all databases	4,238	2.42
Annotated in at least one database	44,672	25.54
Total unigenes	174,903	100

GO database analysis facilitated division of DEGs into Biological Process (BP), Cellular Component (CC) and Molecular Function (MF) categories, and annotated unigenes were further assigned to 49 subcategories (S2 Fig). Analysis of level 2 GO terms showed that in the BP category, the most common annotation terms were cellular process (GO:0009987), metabolic process (GO:0008152), and single-organism process (GO:0044699); in the CC category, the most common annotation terms were cell (GO:0005623), cell part (GO:0044464), macromolecular complex (GO:0032991), organelle (GO:0043226), membrane (GO:0016020), and membrane part (GO:0044425); in the MF category, the most common annotation terms were binding (GO:0005488), catalytic activity (GO:0003824), and transporter activity (GO:0005215).

The 174,903 assembled unigenes were aligned to the COG database to further investigate and classify possible gene functions. In total, 15,241 unigenes were classified into 26 COG categories based on functions according to COG number (S3 Fig). Among the matched sequences, general function prediction only (2,868) represented the largest group of unigenes, followed by signal transduction mechanisms (2,097), posttranslational modification, protein turnover, chaperones (1,833), and translation, ribosomal structure and biogenesis (1,384).

In order to better understand the relationships between DEGs under high salinity conditions, we assigned unigenes based on KEGG pathways (S4 Fig). A total 174,903 unigenes were assigned to Cellular Processes (A), Environmental Information Processing (B), Genetic Information Processing (C), Metabolism (D), and Organismal Systems (E) categories. These five main categories included 32 Hierarchy 2 and 277 KEGG pathways. Among Hierarchy 2 pathways, many unigenes were strongly associated with signal transduction (1,550), translation (1,411), transport and catabolism (902), and carbohydrate metabolism (892).

### Analysis of DEGs

**Fig 2:**
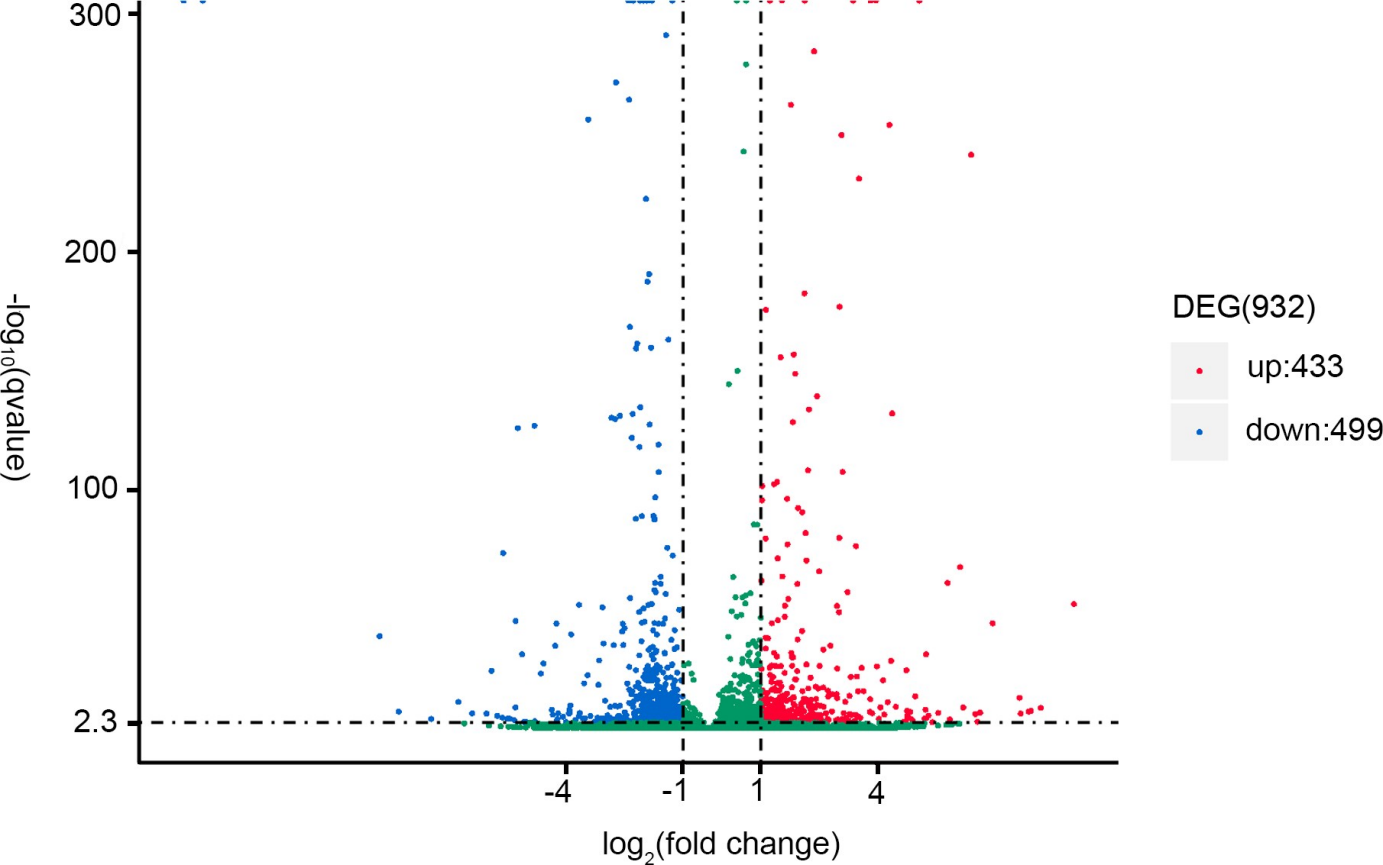
Volcano plot comparing saltwater (SW) and freshwater (FW) treatment groups.

**Fig 3:**
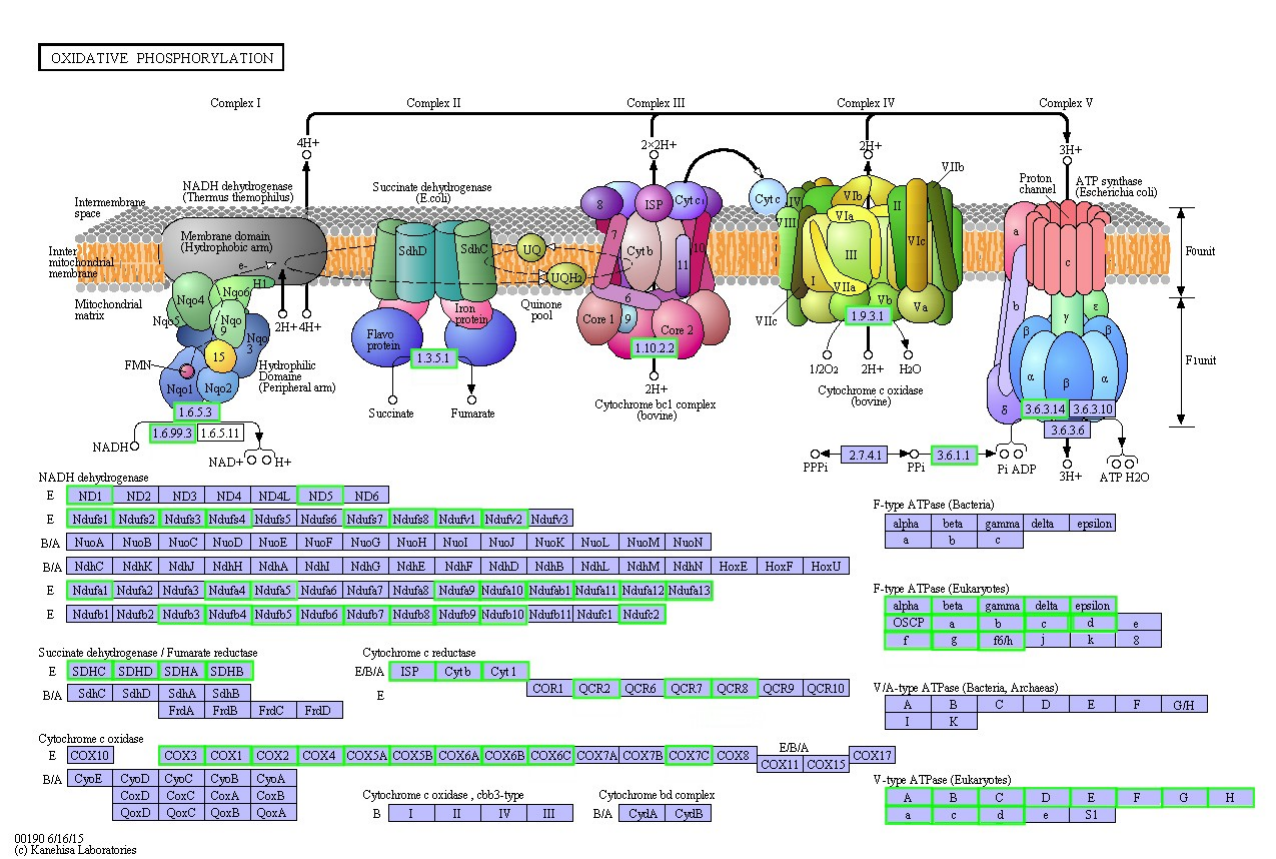
DEGs related to oxidative phosphorylation.

**Fig 4:**
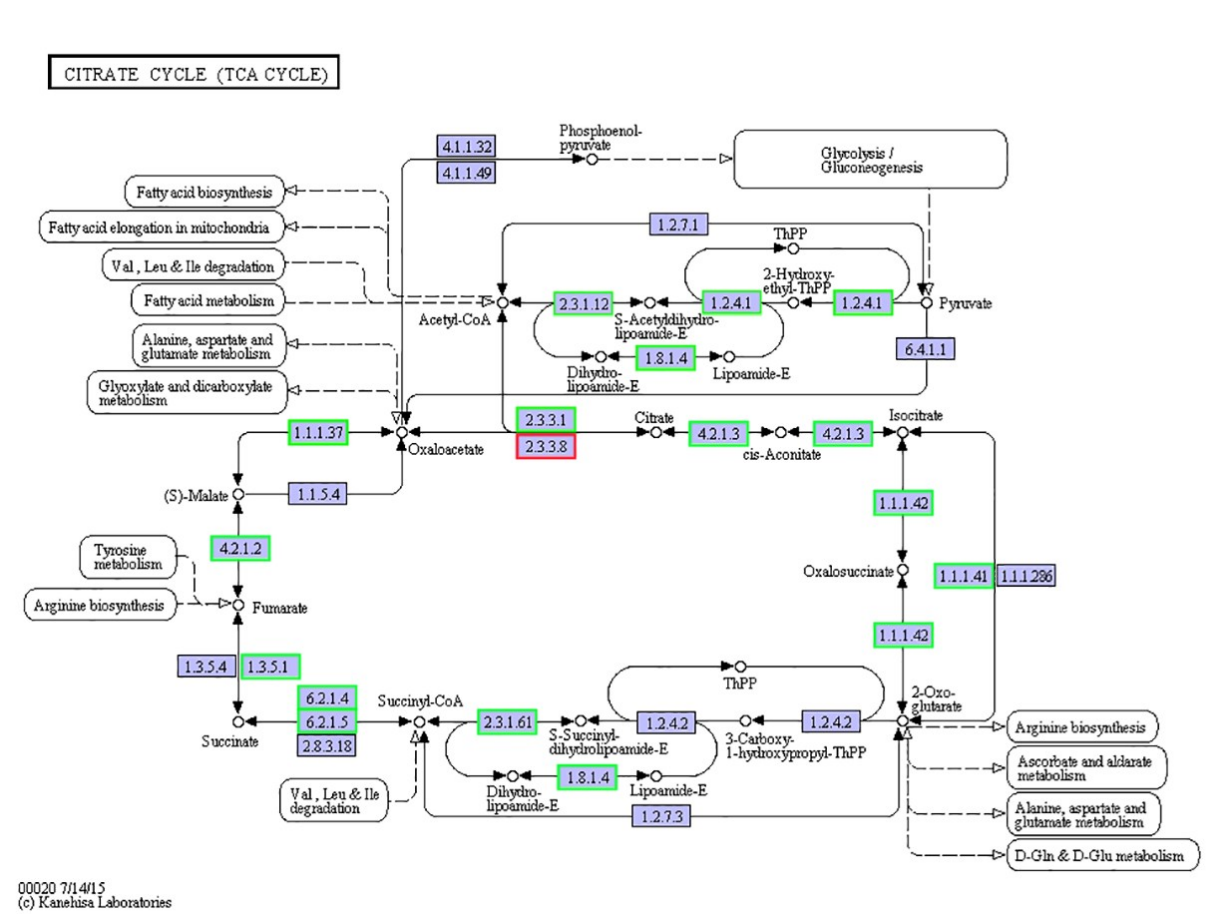
DEGs related to the TCA/citrate cycle.

A total of 932 DEGs were identified from SW and FW gill tissue, of which 433 and 499 were up- and downregulated, respectively (Fig 2). Using the GO database, DEGs were classified according to their biological processes, cellular components and molecular functions (S5 Fig). Based on DEGs of FW vs SW, GO annotations were categorised into 2,021 subcategories within the three major categories. Among biological processes, many DEGs were associated with organophosphate metabolic process (GO:0019637), nucleoside phosphate metabolic process (GO:0006753), nucleoside metabolic process (GO:0009116), ribose phosphate metabolic process (GO:0019693), and nucleoside metabolic process (GO:0009116). Among cellular components, many DEGs were associated with membrane part (GO:0044425), mitochondrium (GO:0005739), organelle envelope (GO:0031967), envelope (GO:0031975), and mitochondrial part (GO:0044429). Among molecular functions, most DEGs were enriched in catalytic activity (GO:0003824) and oxidoreductase activity (GO:0016491). In order to further investigate the relationships between DEGs identified in FW and SW groups, KEGG pathway analysis was performed, and DEGs were categorised into 134 specific KEGG pathways (S6 Fig). Significantly enriched pathways included oxidative phosphorylation (Fig 3), the tricarboxylic acid (TCA) cycle (Fig 4), and collecting duct acid secretion (S7 Fig). The top 13 most enriched KEGG pathways are listed in Table 5. Candidate DEGs potentially associated with salinity adaptation and osmoregulation were categorised into four energy metabolism, transporters, signal transduction and antioxidant pathways based on a combination of enrichment analysis, annotation, and manual literature searches (Table 6).

**Table 5:** Differentiation of pathway analysis of *E*. *sinensis* gill.

Pathway terms	KEGG ID	Number of DEGs Number	Background number^a^	*p*-value
Oxidative phosphorylation	ko00190	75	322	3.52E-27
Cardiac muscle contraction	ko04260	19	72	1.15E-07
Valine, leucine and isoleucine degradation	ko00280	20	110	8.45E-06
TCA/citrate cycle	ko00020	23	155	2.39E-05
Carbon metabolism	ko01200	42	456	0.000145
Rheumatoid arthritis	ko05323	13	79	0.00166
Collecting duct acid secretion	ko04966	10	51	0.002887
Synaptic vesicle cycle	ko04721	14	105	0.005537
Propanoate metabolism	ko00640	10	66	0.014234
Benzoate degradation	ko00362	6	26	0.022755
Fatty acid degradation	ko00071	12	107	0.042323
Caprolactam degradation	ko00930	4	13	0.041195

^a^ Background number: the number of total genes assigned to the pathway

**Table 6:** DEGs potentially associated with salinity adaptation and osmoregulation.

Functional group	Gene name	Gene ID	Fold change (SW/FW)	Gene function	Cellular component
Energy metabolism	Cytochrome c oxidase subunit 2	c119126_g1	-2.4084	Transcription coactivator activity	Mitochondrial inner membrane
	NADH dehydrogenase (ubiquinone) flavoprotein 1	c173489_g1	-2.1231	Oxidoreductase activity, acting on NAD(P)H	Mitochondrial inner membrane
	Glyceraldehyde-3-phosphate dehydrogenase	c169636_g1	-5.0203	Oxidoreductase activity	
	Branched-chain aminotransferase	c157822_g2	-1.8577	Catalytic branched-chain amino acid metabolism	
	Citrate synthase	c134754_g1	-2.6232	Transferring acyl groups	Mitochondrial matrix
	Isocitrate dehydrogenase	c179743_g1	-2.183		Mitochondrial matrix
	Succinate dehydrogenase	c168548_g1	-1.7838	Electron transport	Mitochondrial matrix
	Long-chain-fatty-acid-CoA ligase	c172027_g1	-2.8424	Fatty acid degradation	Mitochondrial matrix
Transporters	Na^+^/K^+^ATPase	c173400_g1	-2.0157	Ion transport	Potassium-exchanging ATPase complex
	V-type H+-transporting ATPase subunit G	c163080_g1	-2.3686	Hydrogen ion transmembrane transporter activity	Membrane
	V-type H+-transporting ATPase subunit F	c170449_g1	-2.2102	Hydrogen ion transmembrane transporter activity	Membrane
	Na^+^-K^+^-2Cl^-^contransporter	c165384_g3	2.4757	Sodium ion transport	Membrane
	Chloride channel 2	c182730_g1	-1.6306	Chloride transport	Membrane
	ATP-binding cassette, subfamily C	c179421_g1	4.3496	Transmembrane transport	Integral component of membrane
Signal transduction	Dopamine receptor D1	c167704_g1	4.0794	G-protein coupled receptor activity	Integral component of membrane
	Synaptotagmin-1	c181649_g2	2.2691	Regulation of neurotransmitter release	Membrane
	Syntaxin-binding protein 1	c179461_g2	1.3491	Regulation of neurotransmitter release	Membrane
	ADP-ribosylation factor 1	c170851_g2	6.1241	Cell vesicle transport process	Membrane
	Phospholipase D1/2	c161757_g1	1.8775	Phospholipid signal transduction	
Antioxidant activity	Cu^2+^/Zn^2+^ Superoxide dismutase	c153237_g1	6.5184	Antioxidant activity	
	Glutathione S-transferase	c163902_g1	1.4387		
	Glucose-6-phosphate 1-dehydrogenase	c178975_g1	1.5714	Oxidation-reduction process	
	Glutathione peroxidase	c153117_g1	-1.2138	Response to oxidative stress	
	Macrophage migration inhibitory factor	c142195_g1	-4.2166	Immunoregulatory	

### qRT-PCR validation of RNA-Seq data

**Fig 5:**
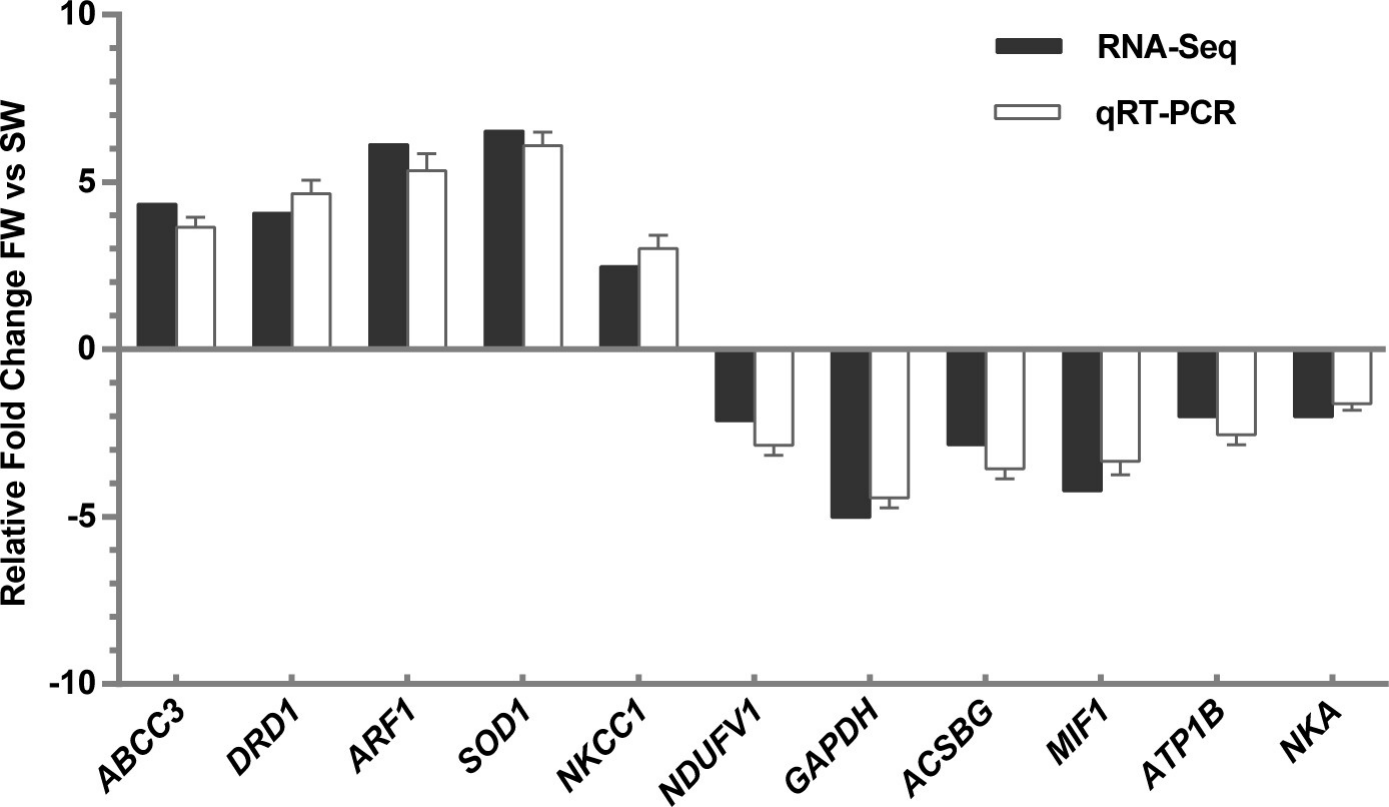
qPCR validation of RNA-Seq data.

To validate our Illumina sequencing results, five upregulated DEGs (ABCC3, DRD1, ARF1, SOD1 and NKCC1) and six downregulated genes (NDUFV1, GAPDH, ACSBG, MIF1, ATP1B and NKA) were selected or verified by qRT-PCR. The results showed that the expression trends of all ten DEGs were consistent with the high-throughput RNA-Seq results, indicating that the sequencing results were reliable (Fig 5).

## Discussion

In recent years, transcriptome analysis technology has developed rapidly, and has been applied to the study of aquatic animals. *E*. *sinensis* is a representative species for analysis of hyperosmotic regulation [5, 30]. In this species, osmoregulatory mechanisms are activated in response to changes in environmental salinity during reproductive migration [31]. To further investigate the potential mechanisms underpinning osmotic regulation and identify osmotic-regulated genes, we compared the transcriptomes of *E*. *sinensis* gill tissue in freshwater (FW; 0 ppt) and seawater (SW; 25 ppt) treatment groups. A total of 932 DEGs were identified, comprising 433 and 499 up- and downregulated genes. We analysed these DEGs with an emphasis on genes related to energy metabolism, ion channel regulation, signal transduction, and oxidative stress.

### Energy metabolism

Osmotic pressure regulation is achieved by several ion transporters and ion transport channels, which require a large amount of energy [32]. However, in our present study, most DEGs related to two important energy metabolism pathways, oxidative phosphorylation and the TCA cycle, were downregulated. Genes related to nicotinamide adenine dinucleotide (NADH) dehydrogenase, ubiquinone cytochrome C reductase, cytochrome C oxidase, and ATP synthase were downregulated. These enzymes are located on the mitochondrial membrane and constitute electron transfer chains that synthesise ATP through biological oxidation [33]. The same trend was found for genes related to the TCA cycle; Citrate synthase (CS), isocitrate dehydrogenase and succinate dehydrogenase were all downregulated. As the first rate-limiting enzyme of the TCA cycle, CS catalyses the condensation of oxaloacetic acid and acetyl coenzyme A to produce citric acid and coenzyme A for ATP production [34]. Isocitrate dehydrogenase catalyses the oxidative decarboxylation of isocitrate to produce α-ketoglutaric acid, CO2 and NADH, providing energy for organisms and biosynthetic precursors. NADH is an important coenzyme in cells that is involved in most oxidation-reduction reactions of sugars, fats and proteins [35]. Succinic dehydrogenase converts succinic acid into fumaric acid, accompanied by the formation of ATP. Unlike other enzymes in the TCA cycle, succinic dehydrogenase is the only enzyme embedded in the mitochondrial inner membrane, and is an important part of the mitochondrial inner membrane [36]. Our current findings appear to contradict those of a previous study in which osmoregulatory processes are accompanied by an increase in energy demand [11], but these results were consistent with those of Zhang et al. (2018) [12]. The process of osmotic pressure regulation in *E*. *sinensis* can also be divided into two stages; in the initial stage (1–3 days), the energy requirement of osmotic pressure regulation increases, hence energy metabolism increases; in the subsequent regulatory stage (at 3 days after transfer), the energy requirement decreases, and energy metabolism returns to normal levels. We speculate that after 144 h of high salinity stress, osmotic pressure regulation in *E*. *sinensis* enters a relatively stable stage, during which the energy required for osmotic regulation is decreased (Fig 1) [37]. Additionally, a salinity of 25 ppt is close to the isosmotic state for *E*. *sinensis* (33 ppt). At the isosmotic point, the energy used by organisms to maintain osmotic pressure is minimal [38]. This results in the downregulation of genes encoding enzymes are related to energy metabolism. Further studies should be performed, including time courses, to probe the link between energy metabolism and osmotic adjustment in *E*. *sinensis*.

### Transporters

The ion transport epithelium in the gill is the main location for osmotic adjustment and ion transport in crustaceans [39]. Ion transport is accomplished mainly by NKA and other ion transport enzymes [40]. Gill epithelium NKA is essential for hyperosmotic crustaceans. It is mainly involved in osmotic regulation and ion regulation, can switch between Na^+^ and K^+^ transport, and can also affect the activity of other enzyme systems related to ionic regulation on the gill epithelium [41]. In our current transcriptome analysis, expression of NKA in the gill filament of the SW group was significantly downregulated compared with the control group. *E*. *sinensis* is a hyperosmoregulator, which means it needs to take up salt from the medium when salinity fluctuates between freshwater and seawater. We conclude that the downregulation of NKA is most likely due to *E*. *sinensis* is close to isosmotic to the seawater (25ppt), which would reduce NKA activity [42, 43].

Another classic ion transporter, NKCC1, was also included among the identified DEGs. NKCC1 is particularly important for regulating ion transport, and belongs to the SLC12A family that includes two subtypes, NKCC1 and NKCC2 [44]. NKCC1 is closely related to the secretion of Cl^−^ in epithelial cells, and is considered the secretion subtype [45]. In the present study, expression of NKCC1 was upregulated in the gill of *E*. *sinensis*, which reflects the activation of chloride secretion. Crabs may accelerate the secretion of Cl^-^ from the body by increasing NKCC1 levels in a high salinity environment, thereby achieving salt and water retention.

Furthermore, the gene encoding V-type H^+^-ATPase (VHA) was downregulated following salinity stress. During osmoregulation in teleosts, VHA is a major ion-regulating enzyme, second only to NKA, which powers the uptake of sodium and chloride ions by excreting protons [46]. Our results imply that VHA may be downregulated in response to high salinity in the surrounding water body. This reflects a reduction in sodium chloride intake by the body.

### Signal transduction

Some studies have shown that neuroendocrine systems can regulate the activity of NKA, VHA, and cystic fibrosis transmembrane conductance regulator in epithelial cells via cAMP-dependent pathways [47, 48, 49]. In the present study, DRD1 was significantly upregulated after salinity stress. Dopamine binds to dopamine receptors and plays an important role in maintaining the balance of ions in kidney [50]. DRD1 enhances the role of cAMP in cells by activating adenylate cyclase [51]. NKA activity can be inhibited by phosphorylation of NKA subunits mediated by protein kinase A [52]. Thus, we infer that under high salinity stress, *E*. *sinensis* might reduce the activity of NKA by upregulating the expression of DRD1, thereby achieving ionic regulation.

In addition, we also found that STXBP1 was upregulated significantly. STXBP1 is a very important protein in synaptic vesicles, which is fixed to the membrane of secretory vesicles via a single transmembrane domain at its N-terminus. It can sense the flow of Ca^2+^ and cause the fusion of vesicles and the release of neurotransmitters [53]. This further indicates that during osmotic adjustment, the neuroendocrine system may regulate ion transport in the gill epithelium through neurotransmitter signalling.

### Antioxidant pathways

Changes in salinity can cause an increase in ROS in fish that may result in oxidative damage if not cleared quickly [54]. Antioxidant mechanisms are the main barrier against oxidative damage in fish. Fish can scavenge ROS and enhance resistance through antioxidant mechanisms [55]. In the present study, various antioxidant genes were included among the identified DEGs, including GST (upregulated), glucose-6-phosphate dehydrogenase (G6PDH) (upregulated), and SOD1 (upregulated). GST is an important detoxifying enzyme in organisms that attaches glutathione to various electrophilic compounds and increases their solubility, thereby facilitating their excretion from cells. GST also possesses glutathione-dependent peroxidase activity and protects the body from endogenous peroxide damage [56]. G6PDH provides the reductive NADH coenzyme for biosynthesis, and produces glutathione, which regulates the redox state of the organism [57]. We speculate that high salinity stress may cause oxidative stress in *E*. *sinensis*, and GST and G6PDH likely play important roles in preventing oxidative damage in crabs.

## Conclusions

The present study demonstrates that brackish water conditions decrease energy metabolism and enhance the antioxidant capacity of Chinese mitten crab. *E*. *sinensis* can mobilise the neural transmission system to alter the activity of ion transport enzymes and osmotic regulation. However, the details of osmotic regulation in *E*. *sinensis* remain unclear, and protein expression should also be analysed to elucidate the mechanisms responsible.

## Supporting information

S1 Fig Unigene transcript length distribution. (TIF)

S2 Fig Unigene GO annotation (level 2). (TIF)

S3 Fig Unigene COG annotation. (TIF)

S4 Fig Unigene KEGG pathway annotation. (TIF)

S5 Fig GO annotation of DEGs in FW vs. SW. (TIF)

S6 Fig SW vs. FW DEG enriched KEGG pathway scatterplot. (TIF)

S7 Fig Collecting duct acid secretion pathway. (TIF)
