# Supplementary figures and images for: Comparative transcriptome analysis reveals osmotic-regulated genes in the gill of Chinese mitten crab (Eriocheir sinensis)
Source: PLoS One. 2019 Jan 10;14(1):e0210469. doi: 10.1371/journal.pone.0210469 (PMC6328174; doi:10.1371/journal.pone.0210469)

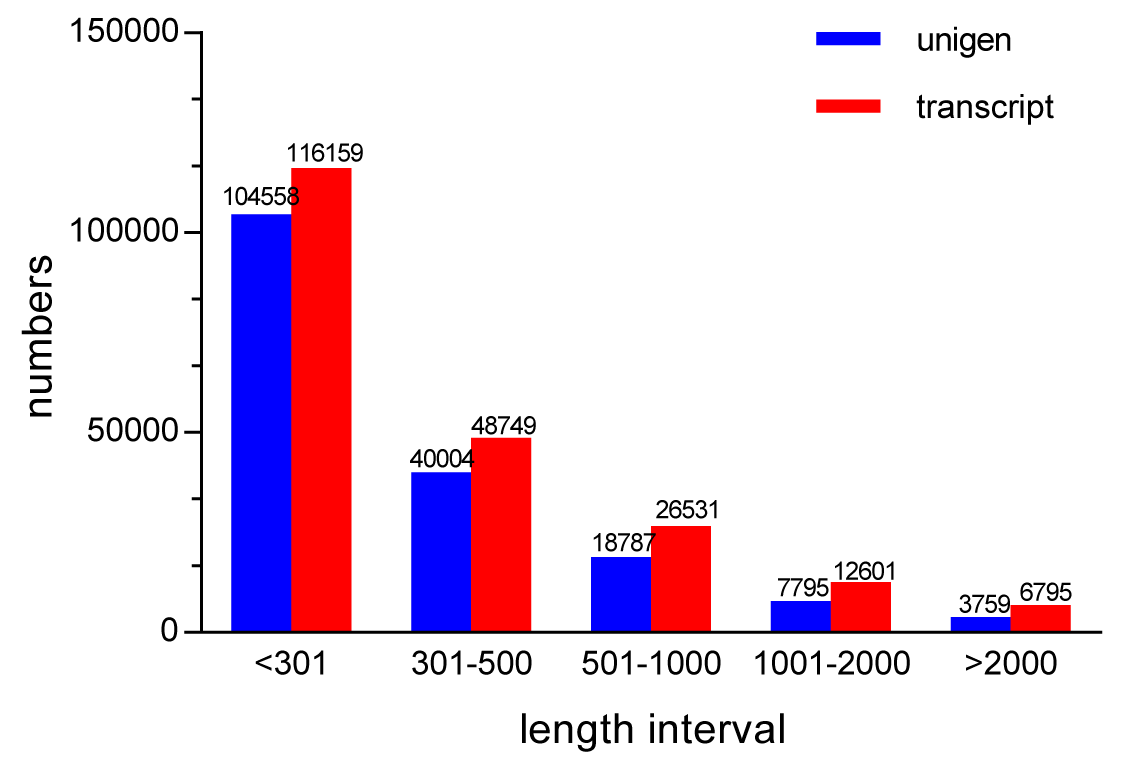

Supplement: S1 Fig — (TIF) [file pone.0210469.s001.tif]

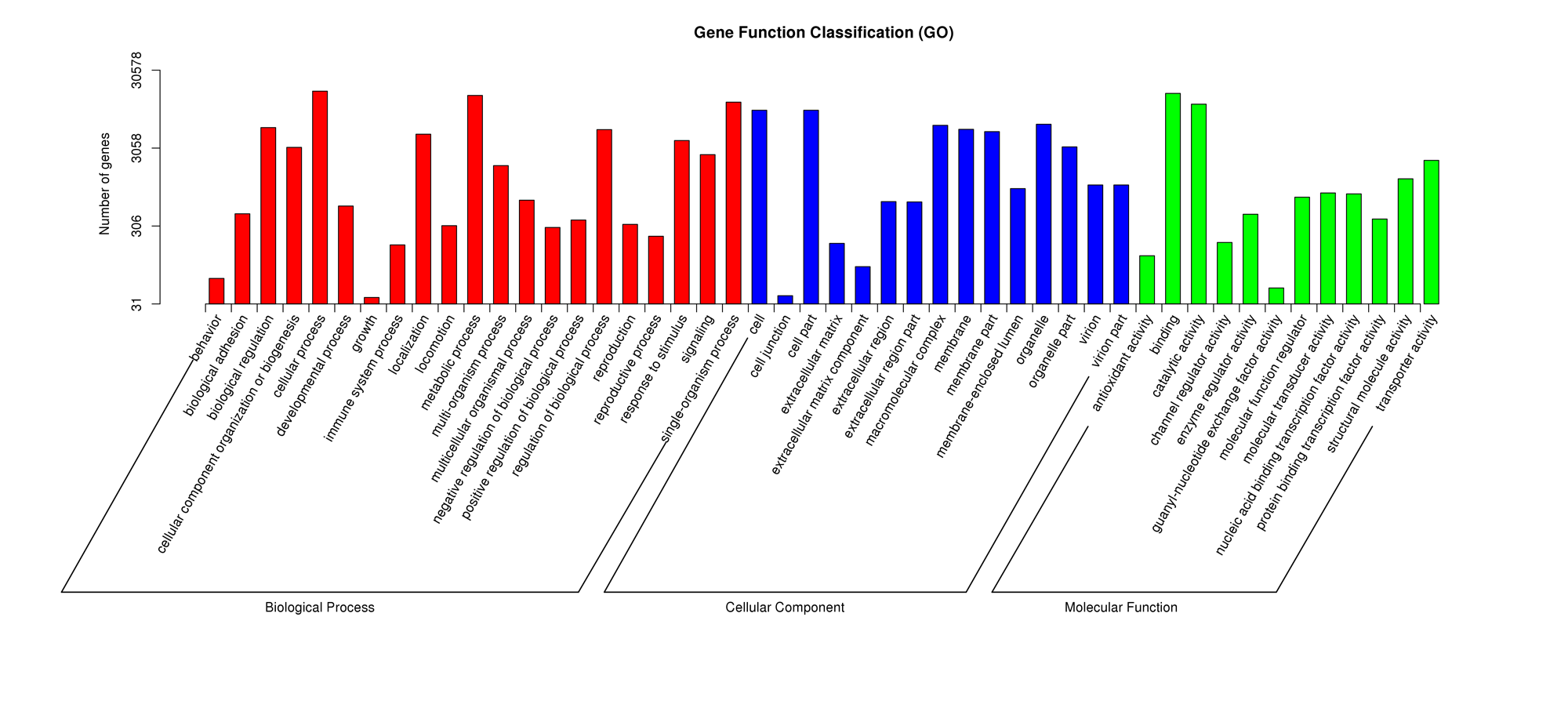

Supplement: S2 Fig — (TIF) [file pone.0210469.s002.tif]

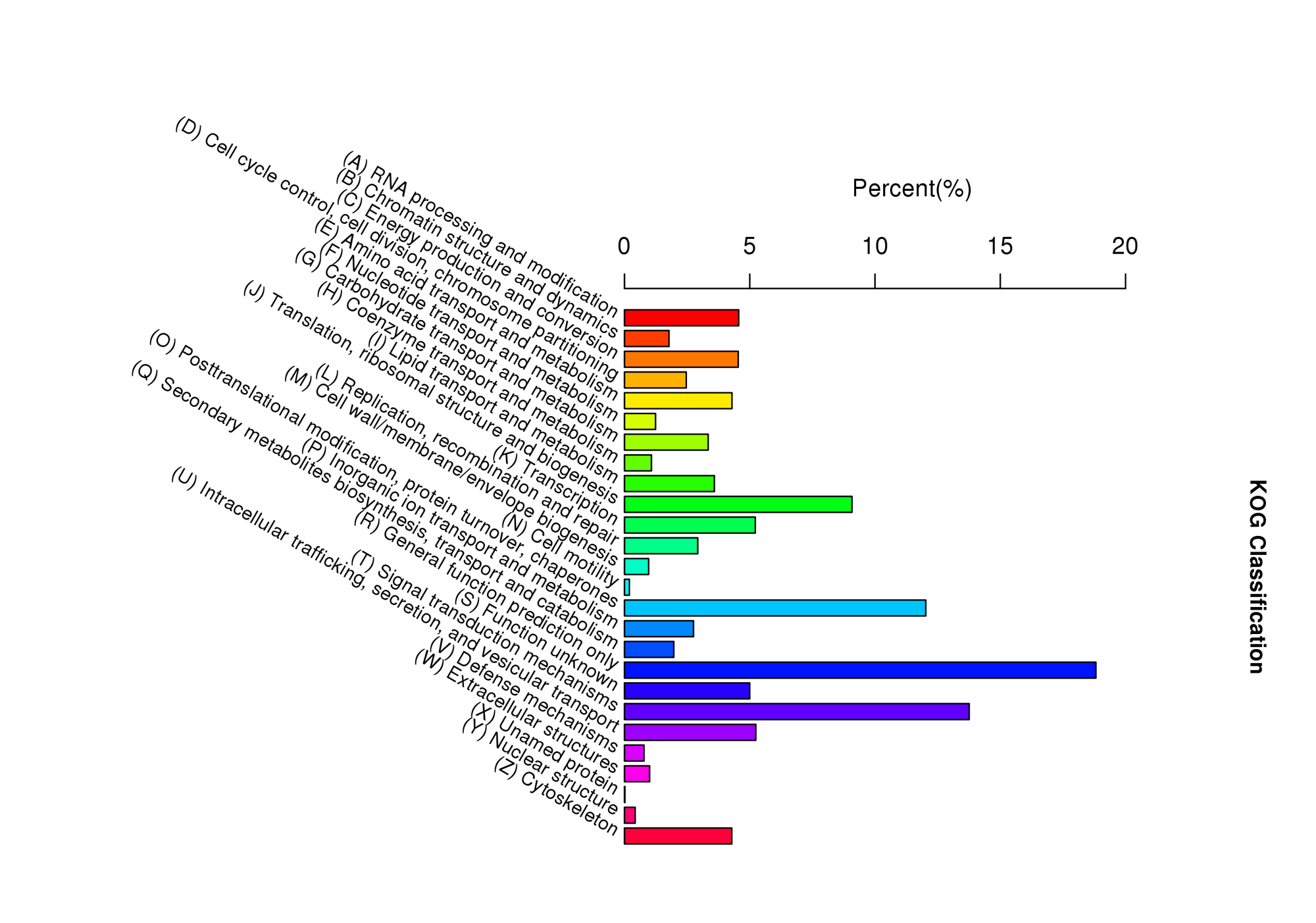

Supplement: S3 Fig — (TIF) [file pone.0210469.s003.tif]

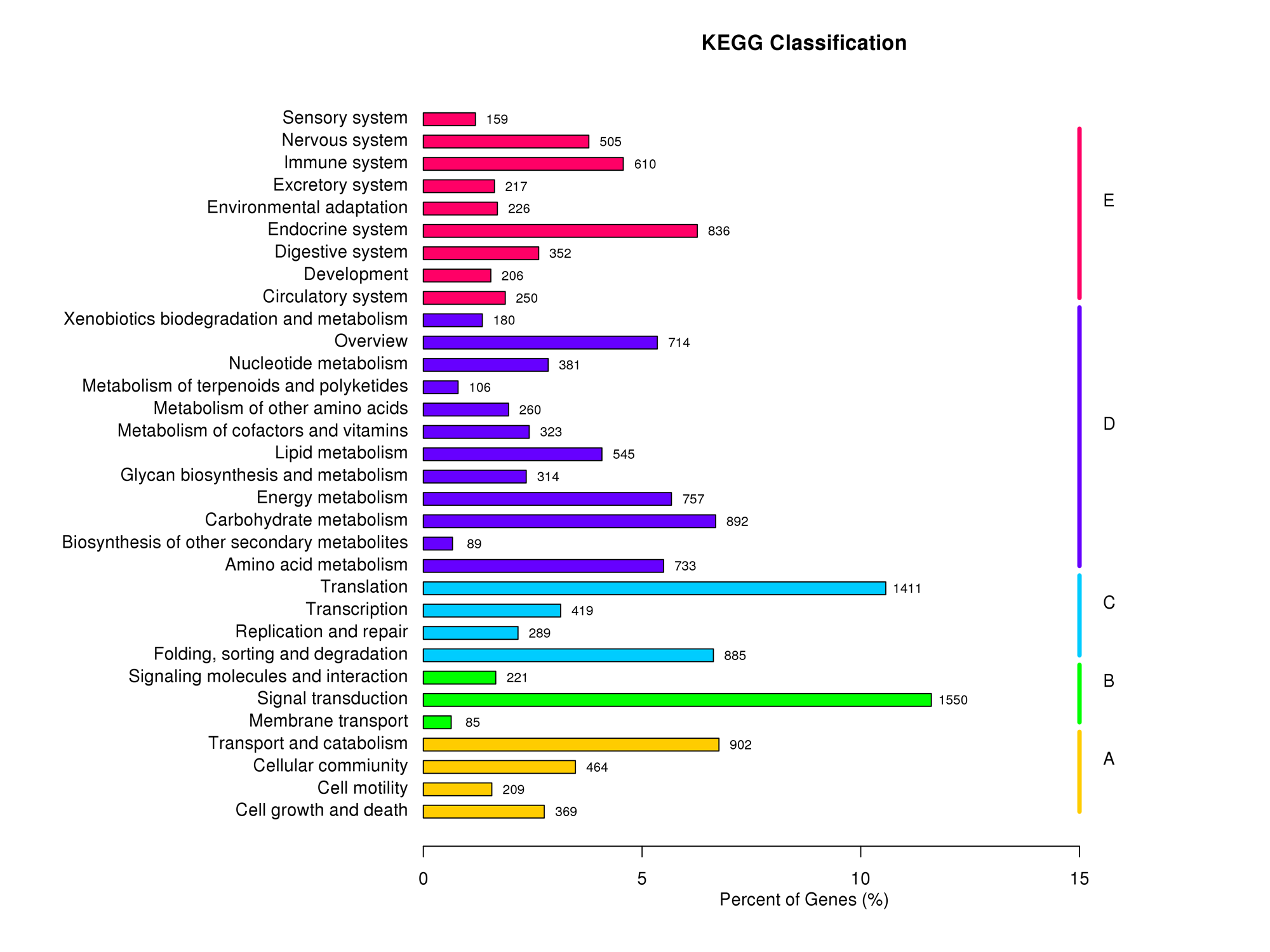

Supplement: S4 Fig — (TIF) [file pone.0210469.s004.tif]

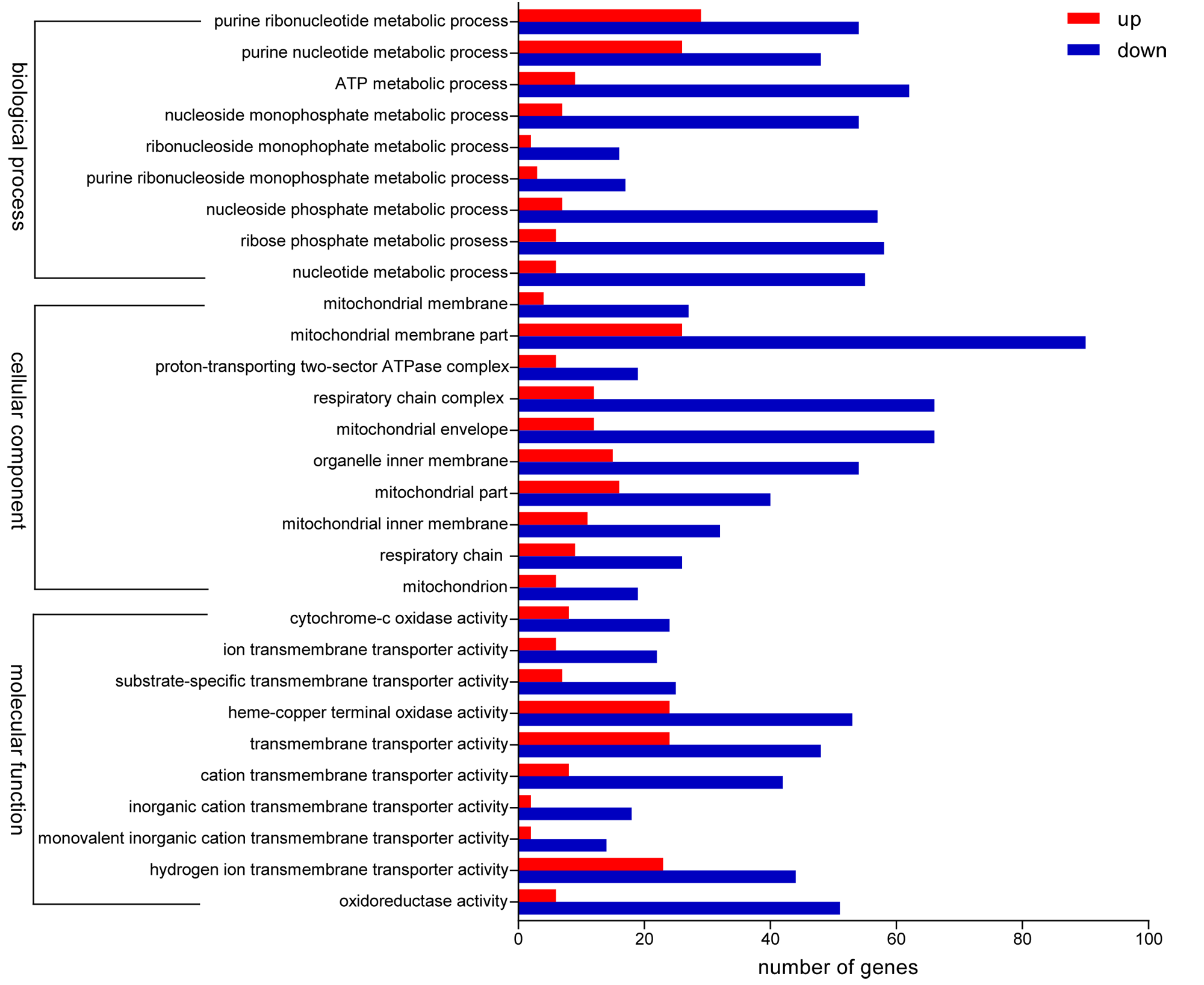

Supplement: S5 Fig — (TIF) [file pone.0210469.s005.tif]

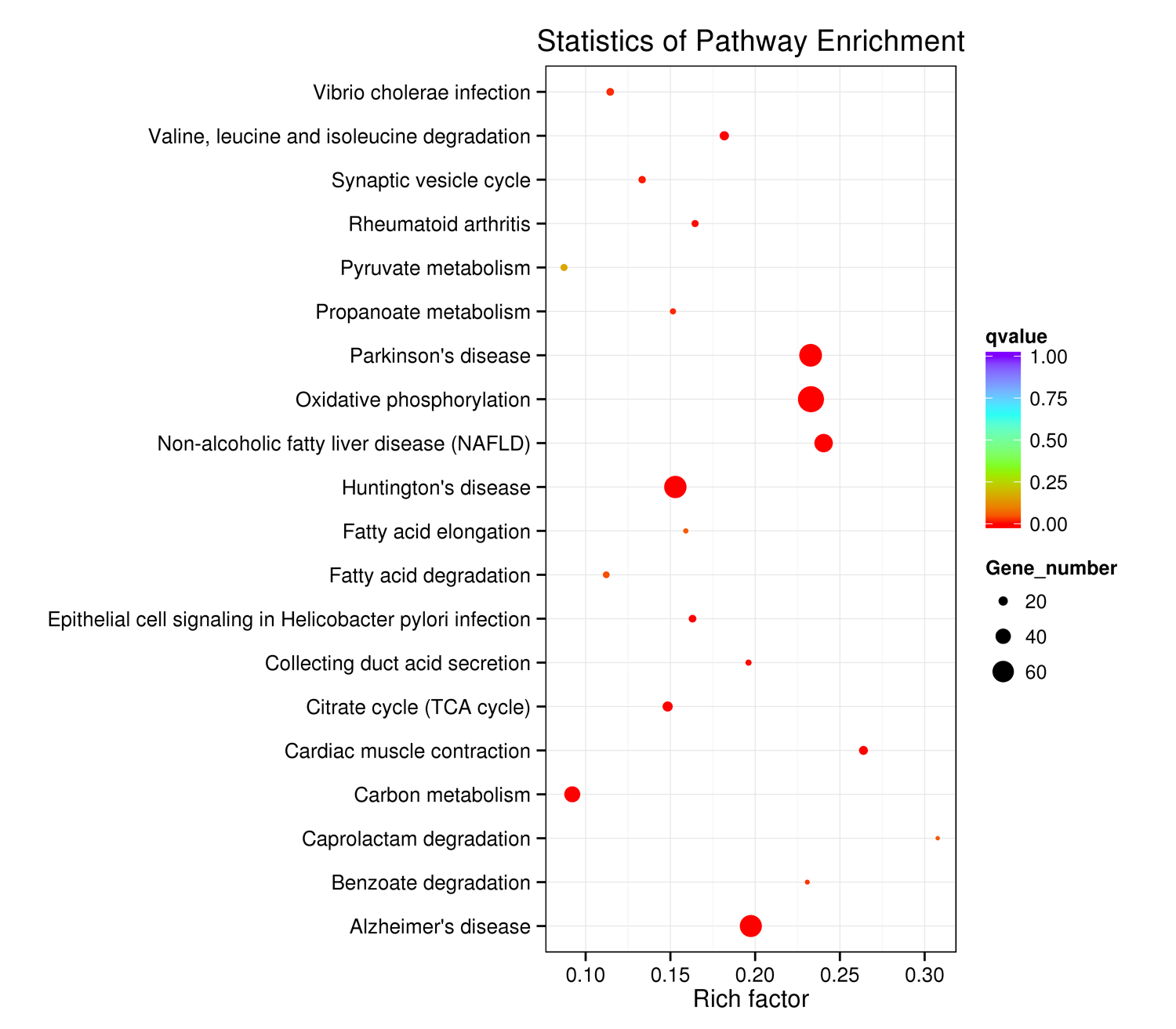

Supplement: S6 Fig — (TIF) [file pone.0210469.s006.tif]

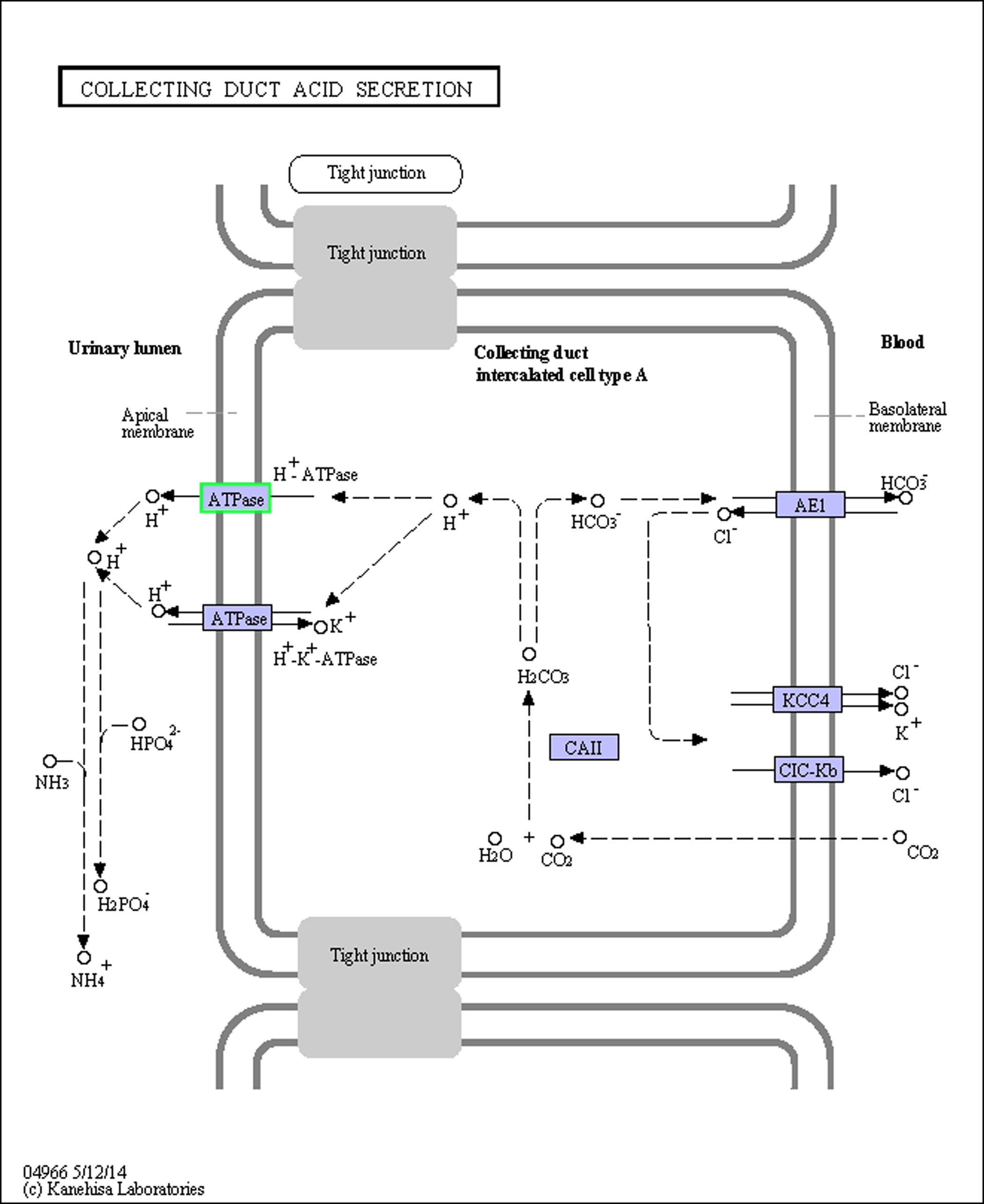

Supplement: S7 Fig — (TIF) [file pone.0210469.s007.tif]
